# LAPTM5 Confers the Resistance to Venetoclax via Promoting the Autophagosome‐Lysosome Fusion in Multiple Myeloma

**DOI:** 10.1111/jcmm.70331

**Published:** 2025-01-03

**Authors:** Yuxiang Li, Jing Bai, Dan Liu, Jinxia Hao, Ruyu Yan, Hongjuan Guo, Yuzhi Huang, Hongtao Yu, Hao Leng, Kecheng Zhou, Minxia Liu

**Affiliations:** ^1^ School of Life Sciences Anhui Medical University Hefei China; ^2^ Department of Biochemistry and Molecular Biology, School of Basic Medical Sciences Anhui Medical University Hefei China; ^3^ Department of Internal Medicine Xi'an Jiaotong University Hospital Xi'an China

**Keywords:** autophagy, drug resistance, LAPTM5, multiple myeloma, venetoclax

## Abstract

Multiple myeloma (MM) is a haematological lymphoid malignancy marked by significant morbidity due to severe complications. Despite advances in targeted therapies, including proteasome inhibitors and the BCL‐2 inhibitor venetoclax, drug resistance frequently occurs, with the underlying mechanisms poorly understood. This study investigates the role of lysosome‐associated protein transmembrane 5 (LAPTM5) in conferring resistance to venetoclax in relapsed MM. Using comprehensive analyses of publicly available databases and experimental validation, we demonstrated that LAPTM5 is upregulated and enhances autophagy in recurrent multiple myeloma cells, which is a key process for cell homeostasis and drug resistance. Mechanistic studies reveal that LAPTM5 facilitates autophagic flux, linking it to the cellular catabolic processes essential for survival under therapeutic stress. Our findings highlight the underexplored functions of LAPTM5 in modulating autophagy and drug resistance, we demonstrate that LAPTM5 confers resistance to venetoclax by enhancing autophagy, suggesting that targeting LAPTM5 may provide new avenues for overcoming treatment challenges. This research underscores the potential function of LAPTM5 as a therapeutic target in improving outcomes in MM treatment.

**Abbreviations**
AML: acute myeloid leukaemia
CLL: chronic lymphocytic leukaemia
LAPTM5: lysosome associated protein transmembrane 5
MM: multiple myeloma
UPS: ubiquitin‐proteasome system

## 1 Introduction

Multiple myeloma (MM) is an aggressive neoplasm of malignant plasma cells that presents considerable challenges due to its propensity to induce various complications, including bone disorders, renal impairment and anaemia [1, 2]. Multiple myeloma (MM) is a malignant plasma cell disorder that accounts for approximately 10% of all hematologic cancers [2]. Despite the advent of targeted pharmacological therapies, such as proteasome inhibitors, immunomodulatory agents and monoclonal antibodies, the emergence of drug resistance in MM cells remains a significant hurdle, leading to relapse following initial treatment [3]. MM cells are characterised by the production of excessive amounts of unfolded or misfolded proteins, which can be detrimental to cellular health. Consequently, the degradation of these aberrant proteins is vital for the survival of MM cells [4]. Two primary pathways are involved in intracellular protein degradation: the ubiquitin‐proteasome system (UPS) and the autophagy‐lysosome pathway [5]. Currently, proteasome inhibition serves as a cornerstone in the therapeutic landscape for MM.

Venetoclax, a selective BCL‐2 inhibitor, antagonises BCL‐2 family anti‐apoptotic proteins by mimicking the BH3 domain of pro‐apoptotic proteins [6]. Its therapeutic efficacy has been notably recognised in haematological malignancies, particularly in chronic lymphocytic leukaemia (CLL), acute myeloid leukaemia (AML) and MM. Venetoclax has shown promising efficacy in the treatment of MM, especially in patients harbouring the translocation t(11;14), which is among the most prevalent chromosomal abnormalities. The pronounced dependency on Bcl‐2 makes t(11;14) MM uniquely susceptible to venetoclax, a selective Bcl‐2 inhibitor [2, 7]. However, it is noteworthy that venetoclax is not a curative agent for MM, with treatment impeded by inevitable disease relapse over time [8]. Recent investigations have identified recurrent mutations in BCL2, BAX and the cell cycle regulators BTG1 and CDKN2A as contributors to venetoclax resistance [9]. Although genetic determinants of venetoclax resistance have been explored, the role of autophagy in modulating this resistance remains inadequately understood.

Lysosomes, as cellular organelles responsible for the degradation and recycling of macromolecules and damaged organelles, have increasingly been recognised for their critical roles in regulating nutrient metabolism and maintaining energy balance [10]. Emerging research underscores the involvement of lysosomes in facilitating drug resistance through various mechanisms, including lysosomal retention and the regulation of intracellular membrane transport [11]. Nonetheless, the role of lysosomes in mediating drug resistance in MM is largely underexplored, despite preliminary studies suggesting a potential impact of lysosomal proteins on resistance mechanisms [12]. Therefore, further investigation into the function of lysosomes in drug resistance is imperative for the development of effective treatment strategies for MM. Autophagy, a fundamental lysosomal function, is a central physiological process that sustains cellular homeostasis and significantly influences drug resistance in MM and other malignancies [13]. Under adverse conditions such as hypoxia, oxidative stress, pathogen invasion, or nutrient scarcity, autophagy is activated as a self‐protection mechanism to enhance cell survival [10, 14]. This process has been implicated in the pathogenesis of numerous diseases and plays a crucial role in shaping cellular drug resistance [15].

The LAPTM family, comprising crucial lysosomal membrane proteins, plays a significant role in maintaining lysosomal function and regulating cellular processes. This family includes LAPTM4A, LAPTM4B and LAPTM5, all localised to lysosomes and late endosomes [16]. LAPTM4A was critical in the biosynthesis of globotriaosylceramide [17, 18, 19]. Most of LAPTM4B investigations focus on cancer research, several cohort studies reported LAPTM4B is upregulated in various tumours, including NSCLC [20], breast cancer [21] and leukaemia [22]. LAPTM4B stimulates cancer proliferation [23], autophagy [24] and resistance to chemotherapeutic drugs [25, 26]. We previously demonstrated that LAPTM4B regulates cytoskeleton arrangement [27], controls the recycle of integrin beta1 [28], suppresses ferroptosis [29] and is involved in regulating cell metabolism [30, 31, 32]. However, studies assessing LAPTM5 function in cancer pathology are still scarce. LAPTM5 regulates the inflammatory response [33]. LAPTM5 has been shown to induce lysosomal cell death by destabilising lysosomes and promoting proliferation and invasion in breast cancer, facilitating lung‐specific metastasis and triggering mitochondrial apoptosis [34]. Moreover, LAPTM5 is a driver of resistance to lenvatinib in hepatocellular carcinoma [35]. However, the role of LAPTM5 in mediating resistance in MM, and the detailed mechanistic insights, remains poorly investigated.

In this study, we conducted an analysis of publicly accessible databases to explore the expression and potential clinical significance of LAPTM5 in MM. Our findings reveal that LAPTM5 is highly expressed in relapsed MM and plays a role in the positive regulation of cellular catabolic processes within this context. Autophagy, a crucial cellular metabolic pathway reliant on the fusion between autophagosomes and lysosomes [36], is shown to be significantly promoted by LAPTM5. Furthermore, mechanistic studies indicate that LAPTM5 is crucial for both autophagy and drug resistance, as autophagy itself contributes to mediating resistance to pharmacological agents [15]. By integrating results from cellular assays, patient tissue analyses and extensive cancer databases, this study provides comprehensive evidence emphasising the clinical relevance of LAPTM5‐regulated autophagy in venetoclax resistance, suggesting its potential as a therapeutic target for addressing MM progression.

## 2 Materials and Methods

### 2.1 Human Cell Lines

The Multiple myeloma cell line OPM2 and 8226 was purchased from Shanghai Jinyuan Biotechnology Co. Ltd. It was cultured in RPMI‐1640 medium, supplemented with 10% fetal bovine serum (FBS) and 1% Penicillin–Streptomycin Solution, under standard conditions of 37°C with an atmosphere of 5% CO_2_. The OPM2/VR cells and 8226/VR cells underwent treatment with venetoclax at incremental concentrations ranging from 50 nM to 1000 nM, with the drug concentration being doubled every 5 days.

Cells with knockdown of LAPTM5 and ATG5 were established utilising short hairpin RNA (shRNA) technology. Specifically, coding sequences for LAPTM5 (Gene ID: 7805) and ATG5 (Gene ID: 9474) were analysed to inform the design of effective targeting sequences (accessible via https://rnaidesigner.thermofisher.com/rnaiexpress/). Following this, cells were transfected with plasmids encoding the shRNA and subsequently selected in culture media containing puromycin at a concentration of 2 μg/mL for a duration of 48 h. The resulting knockdown cells were isolated and confirmed through western blotting.

To generate a stable overexpression cell line for LAPTM5, OPM2 cells and 8226 cells were transfected with LAPTM5 using the Hieff Trans Liposomal Transfection Reagent. These cells were then cultured in a medium containing 2 μg/mL puromycin until a resistant cell pool was established.

### 2.2 Reagents, Antibodies and shRNAs

The Hieff Trans Liposomal Transfection Reagent (Cat#40802) was obtained from Yeasen. ABT‐737(Cat#HY‐50907) and Navitoclax (Cat#HY‐10087) were ordered from MCE company. The rabbit anti‐LAPTM5 antibody was sourced from Biorbyt (Cat#orb593045), while the rabbit monoclonal anti‐ATG5 antibody was acquired from Huabio (Cat#ET1611‐38). LAPTM4B antibody was ordered from ATLAS Antibodies (Cat# AMAb91356), Bcl‐2 antibody from Abmart (Cat#T40056). The rabbit polyclonal anti‐LC3 antibody, also from Proteintech (Cat#14600‐1‐AP), and the rabbit monoclonal anti‐p62 antibody, obtained from Huabio (Cat#HA721171), were utilised in the study. Additional antibodies included the GAPDH antibody from Proteintech (Cat#60004‐1‐lg), as well as the α‐tubulin (Cat#66031‐1‐lg) and β‐actin (Cat#66009‐1‐lg) antibodies, Caspase 3 (Cat#19677‐1‐AP) also from Proteintech. Secondary antibodies, including Goat Anti‐Mouse IgG (H + L)‐HRP (Cat#1706516) and Goat Anti‐Rat IgG (H + L)‐HRP (Cat#1706515), were procured from BioRad.

All shRNA in our research are listed in Table 1.

**TABLE 1:** Sequences of shRNAs targeting LAPTM5 and ATG5.

shRNA	Sequence	
shLAPTM5	Forward:	CCGGGCCTTCATCACTGTCCTTATCCTCGAGGATAAGGACAGTGATGAAGGCTTTTTG
	Reverse:	AATTCAAAAAGCCTTCATCACTGTCCTTATCCTCGAGGATAAGGACAGTGATGAAGGC
shATG5	Forward:	CCGGGCAGTGGCTGAGTGAACATCTCTCGAGAGATGTTCACTCAGCCACTGCTTTTTG
	Reverse:	AATTCAAAAAGCAGTGGCTGAGTGAACATCTCTCGAGAGATGTTCACTCAGCCACTGC

The siRNAs targeting LAPTM4B were utilised in a previous study [27], specifically LAPTM4B siRNA1 (GGAUCAGUAUAACUUUUCATT) and LAPTM4B siRNA2 (CCUACCUGUUUGGUCCUUATT). LAPTM4B SiRNAs, other with the Control siRNA, were ordered from Ribobio.

### 2.3 Quantitative‐PCR

For the assessment of mRNA expression, total RNA was extracted from the cells utilising the RNA isolator Total RNA Extraction Reagent (Vazyme, Cat#R401‐01). Complementary DNA (cDNA) was synthesised employing the Hifair V One‐Step RT‐gDNA Digestion SuperMix for qPCR (Yeasen, Cat#11141ES60), utilising the StepOne Real‐Time PCR System (Applied Biosystems, Foster City, CA, USA). Quantitative reverse transcription PCR (qRT‐PCR) was then performed with the Hieff qPCR SYBR Green Master Mix (Yeasen, Cat#11203ES03) in accordance with the manufacturer's instructions.

All primers in our research are listed in Table 2.

**TABLE 2:** Primer sequences for LAPTM5, ATG5 and GAPDH.

Gene	Forward (5′‐3′)	Reverse (5′‐3′)
LAPTM5	CTTCAATGTCCGCATCGCAA	AGGTCAGCGATCCTGAGGTAG
GAPDH	GAAGGTGAAGGTCGGAGTC	GAAGATGGTGATGGGATTTC
ATG5	AAAGATGTGCTTCGAGATGTGT	CACTTTGTCAGTTACCAACGTCA

### 2.4 Western Blotting

For the quantification of cellular protein levels, cells were washed with ice‐cold phosphate‐buffered saline (PBS) and lysed in RIPA lysis buffer (Beyotime, Cat#P0013B). Following a 30‐min lysis period on ice, the cell lysates were subjected to boiling at 100°C for 10 min. Equal quantities of protein were then resolved via 12% SDS‐PAGE and transferred onto a nitrocellulose membrane (Millipore, Cat#HATF00010). The membranes were blocked for 1 h at room temperature with 5% non‐fat milk in TBS containing 0.1% Tween‐20 (TBST) and subsequently incubated with primary antibodies overnight at 4°C. After four washes with TBST, the membranes were incubated with secondary antibodies for 1 h at room temperature. Following additional washes, the membranes were treated with ECL Clarity (Biosharp, Cat#BL523A). Quantification of protein levels was performed by normalising to an internal control protein using ImageJ.

### 2.5 Cell Viability Assay

Stably transfected OPM2 or OPM2/VR cells, as well as stably transfected 8226 or 8226/VR cells, were seeded at 1 × 10^4^ cells per well in 96‐well plates and cultured in the presence of various concentrations of Venetoclax (0, 5 and 10 μmol/L) for 48 h. Subsequently, CCK‐8 reagent (Biosharp, Cat# BS350B) was introduced (10 μL per well), and the plates were incubated for an additional hour. Absorbance at 450 nm was measured using a Spark multifunctional microplate reader (Tecan Spark, USA). The cell survival rate was calculated using the formula: cell viability (%) = [(administration group A—negative control group A)/(non‐administration group A—negative control group A)] × 100%. The relative survival curve was generated utilising GraphPad Prism version 6.0 (GraphPad Software, CA, USA).

### 2.6 Apoptosis Detection by Flow Cytometry

Stably transfected OPM2 and OPM2/VR cells, as well as stably transfected 8226 or 8226/VR cells, were plated in 12‐well plates at 2 × 10^5^ cells per well and cultured in the presence of varying concentrations of Venetoclax (0, 5, or 8 μmol/L) for 48 h. The apoptotic rates of multiple myeloma (MM) cells were assessed through flow cytometry (Cytoflex S, Beckman) utilising an Annexin‐V/PI apoptosis kit (BestBio, Cat# BB‐4101).

### 2.7 Isolation of Peripheral Blood Mononuclear Cells (PBMCs)

PBMCs were isolated by Human peripheral lymphocyte separation kit (Meilunbio, Cat#PWL100) according to the instructions. Briefly, fresh whole blood from four donors, collected with an anticoagulant, was diluted with an equal volume of phosphate‐buffered saline (PBS). An appropriate volume of separation solution was added to a centrifuge tube, and the diluted blood was carefully layered above the separation solution to maintain a distinct interface between the two layers. The sample was then centrifuged at 800 *g* for 30 min at room temperature using a horizontal rotor.

Following centrifugation, the white buffy coat layer, which formed between the plasma and the separation solution, was collected. This layer, containing the PBMCs, was carefully aspirated into a clean 15 mL centrifuge tube and washed with 10 mL of PBS. The suspension was centrifuged at 250–300 *g* for 10 min, and the supernatant was discarded. The cell pellet was resuspended in 10 mL of PBS, and a second centrifugation step was performed at 250–300 *g* for 10 min. After discarding the supernatant, the final PBMC suspension was obtained for further analysis.

### 2.8 Analysis of Autophagic Flux

To evaluate autophagic flux, mCherry‐GFP‐LC3 lentivirus transfection was utilised for the marking and monitoring of LC3. MM cells were transfected with a tandem fluorescent mCherry‐GFP‐tagged adenovirus for 48 h. Images were acquired using confocal fluorescence microscopy, where yellow puncta (representing the merging of GFP and mCherry signals) indicated early autophagosomes, while red puncta (indicating mCherry signal alone) represented late autolysosomes. The evaluation of autophagic flux was based on the colour change of the GFP/mCherry signals.

### 2.9 Bioinformatics Analysis

To identify lysosomal membrane proteins associated with multiple myeloma recurrence, we performed an analysis of the COMMPASS (Clinical Outcomes in Multiple Myeloma: Patient‐derived Analysis of Survival and Study) datasets. The resulting data were summarised and visualised as heatmaps, highlighting 42 lysosomal membrane proteins. Next, we examined the expression of LAPTM5 protein in paired diagnostic and recurrent samples from the public COMMPASS database. Statistical analysis was performed using a paired *t*‐test to evaluate expression differences.

Subsequently, we utilised the FIMM (Finnish Institute for Molecular Medicine) database to identify genes co‐expressed with LAPTM5 and conducted Gene Ontology (GO) enrichment analysis to explore the biological processes associated with LAPTM5 and its co‐expressed genes. The heatmap representation was used to display the top 20 genes from the FIMM database that were either positively or negatively correlated with LAPTM5. Finally, potential co‐expression and physical interaction partners of LAPTM5 were predicted using the STRING database, which led to the identification of several key interacting proteins, including members of the LAPTM family.

### 2.10 Statistical Analysis

All data are presented as mean ± SEM derived from a minimum of three independent experiments. Statistical significance was determined using the t‐test for pairwise comparisons, with a significance threshold set at *p* < 0.05.

## 3 Results

### 3.1 Expression and Potential Roles of LAPTM5 in Multiple Myeloma Unveiled

**FIGURE 1:**
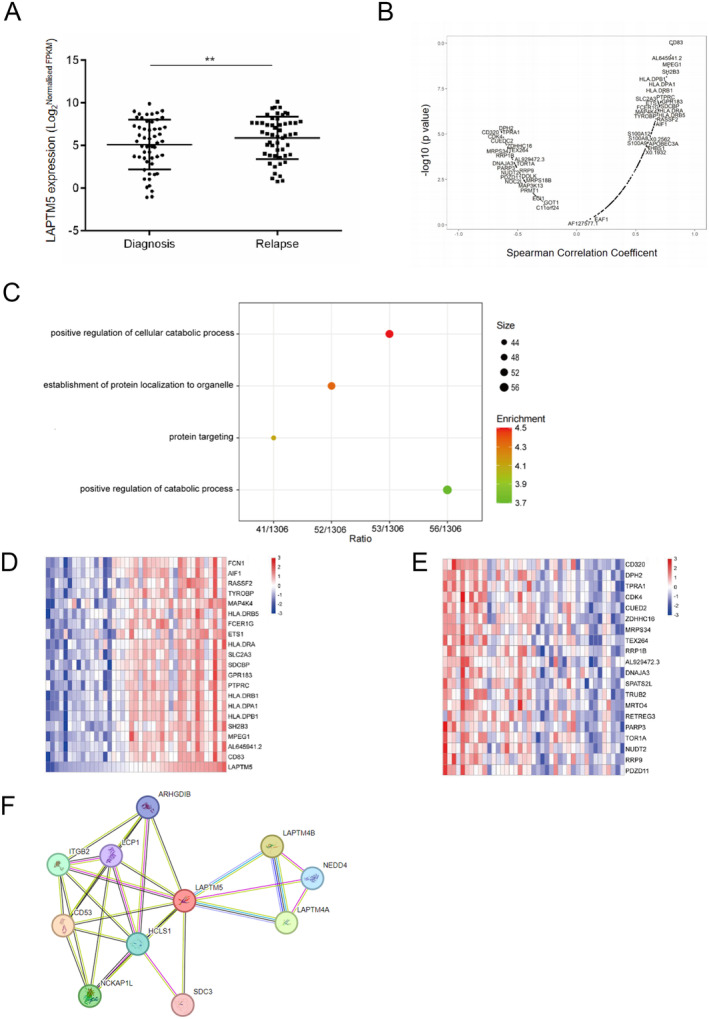
Upregulation of LAPTM5 Levels in Relapsed Multiple Myeloma Patients. (A) A scatterplot illustrating the elevated expression of LAPTM5 in paired diagnostic and relapsed samples from patients within the COMMPASS database, with statistically significant results derived from a paired *T*‐test (**p < 0.01). (B) Identification of genes co‐expressed with LAPTM5. (C) Overview of biological metabolic processes associated with LAPTM5 co‐expressed genes, based on data from the FIMM database. (D) Genes positively correlated with LAPTM5 expression noted in the FIMM database. (E) Genes negatively correlated with LAPTM5 expression confirmed in the FIMM database. (F) Depiction of the LAPTM5 protein interaction network.

Understanding the intricate mechanisms underlying drug resistance in multiple myeloma (MM) is crucial to develop effective therapeutic strategies. Here, we conducted a comprehensive investigation into the expression level of lysosomal membrane protein in MM. Through a combined analysis of GSE6477, GSE9782 and GSE161801, we identified 42 lysosomal membrane proteins associated with the MM relapse, interestingly, we observed that LAPTM5 exhibited a significant difference in expression between diagnostic and relapsed MM patients (Figure S1A). The Peripheral Blood Mononuclear Cells (PBMCs) are routinely used as the control cells of MM [37, 38], we therefore isolated PBMCs from four healthy donors. Our western blot analysis showed that LAPTM5 protein levels are elevated in MM cell lines compared to PBMCs (Figure S1B), further supporting the relevance of LAPTM5 in MM pathophysiology. Given the clinical challenge presented by drug resistance in MM, we focused on the expression levels of LAPTM5 in both MM and paired relapse samples. Our results revealed an upregulation of LAPTM5 expression in relapsed cases compared to the diagnosis cases (COMMPASS dataset) (Figure 1A). To elucidate the functional implications of LAPTM5 and to identify co‐expressed genes, we mined the Finnish Institute of Molecular Medicine (FIMM) database. Applying clustering techniques, we identified gene sets exhibiting co‐expression with LAPTM5 (Figure 1B).

To further investigate the biological processes associated with LAPTM5 and its co‐expressed genes, we conducted Gene Ontology (GO) enrichment analysis. Our findings highlighted significant enrichment in genes implicated in the positive regulation of cellular catabolic processes (Figure 1C). Among the top 20 genes with positive and negative correlations to LAPTM5, we identified CDK4 and ETS1, known to be involved in drug resistance, alongside SDCBP and TEX264, which are associated with autophagy (Figure 1D,E). Next, we predicted the potential co‐expression/physical interaction proteins of LAPTM5 through the STRING website and identified several key proteins as well as LAPTM family proteins that may interact with LAPTM5 (Figure 1F). Collectively, our analysis elucidates the upregulated expression of LAPTM5 in relapsed MM and the possible roles in mediating drug resistance. The enrichment of LAPTM5 and its co‐expressed genes in cellular catabolic processes suggests the critical functions of this lysosomal membrane protein in MM pathogenesis.

### 3.2 LAPTM5 Is Required for the Developing Resistance to Venetoclax

**FIGURE 2:**
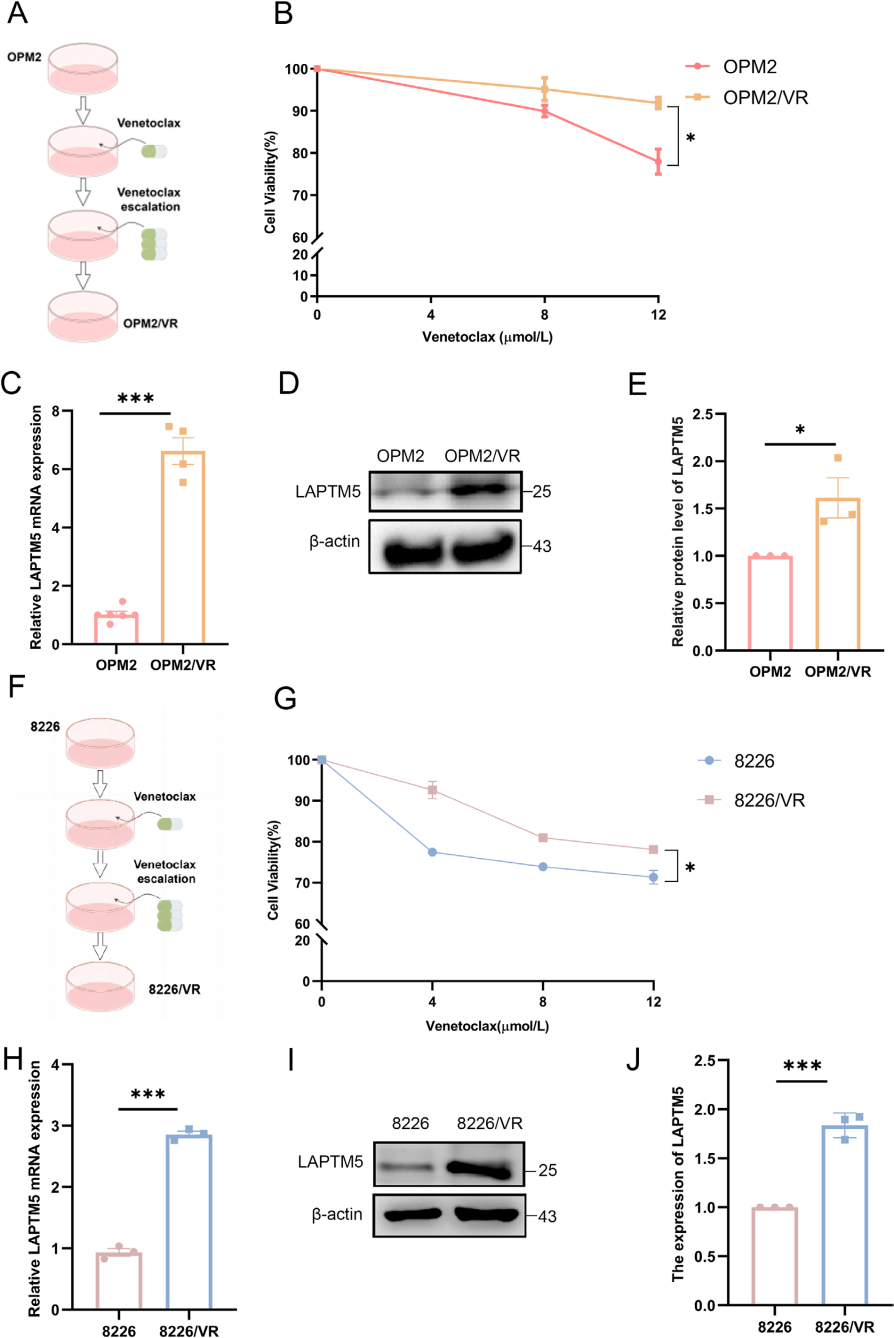
Upregulation of LAPTM5 in Venetoclax‐Resistant Multiple Myeloma Cell Lines. (A) Flowchart detailing the development of venetoclax‐resistant OPM2 cells (OPM2/VR), wherein venetoclax was administered, doubling the drug concentration at intervals until reaching 1 μM. (B) Treatment of OPM2 and OPM2/VR cells with various concentrations of venetoclax (0, 8, 12 μM) for 48 h. Cell viability was assessed via the CCK‐8 assay, with data expressed as mean ± SEM from a paired *T*‐test (*p < 0.05). (C) Analysis of LAPTM5 expression levels via qPCR, presented as mean ± SEM and normalised to ‘WT’. Data were obtained using a paired *T*‐test (***p < 0.001). (D) Western blot analysis reaffirming LAPTM5 gene expression in OPM2/VR cells. (E) Measurement of LAPTM5 protein levels through Western blotting, with quantification from three experiments presented as mean ± SEM and normalised to ‘OPM2’ (paired *T*‐test, *p < 0.05). (F) Flowchart illustrating the generation of venetoclax‐resistant 8226 cells (8226/VR), with drug administration following the aforementioned protocol. (G) Evaluation of cell viability in 8226 and 8226/VR cells treated with venetoclax (0, 8, 12 μM) for 48 h, analysed via CCK‐8 and presented as mean ± SEM from a paired *T*‐test (*p < 0.05). (H) Expression levels of LAPTM5 were also evaluated using qPCR and presented as mean ± SEM, normalised to ‘WT’, with statistically significant results from a paired T‐test (***p < 0.001). (I) Western blot assessment of LAPTM5 expression in 8226/VR cells. (J) Determination of LAPTM5 protein levels through Western blotting, with quantification from three independent experiments as mean ± SEM and normalised to ‘8226’ (paired *T*‐test, ***p < 0.001).

The observed elevation of LAPTM5 expression in relapsed MM prompted us to investigate its role in mediating drug resistance. To achieve this, we established a venetoclax‐resistant MM cell line by subjecting OPM2 cells to gradually increasing concentrations of venetoclax (initiating at 50 nM and escalating to 1000 nM) (Figure 2A). Cell viability assays confirmed the successful generation of venetoclax‐resistant OPM2 cells (designated OPM2/VR) (Figure 2B). Notably, the Quantitative polymerase chain reaction (q‐PCR) and western blotting demonstrated a significant upregulation of LAPTM5 in the OPM2/VR cells (Figure 2C–E). To exclude cell‐specific effects, we generated another resistant cell line named 8226/VR using the same methodology (Figure 2F,G), which likewise exhibited elevated LAPTM5 expression compared to the parental cells (Figure 2H–J). These results suggest a potential role for LAPTM5 in mediating venetoclax resistance.

**FIGURE 3:**
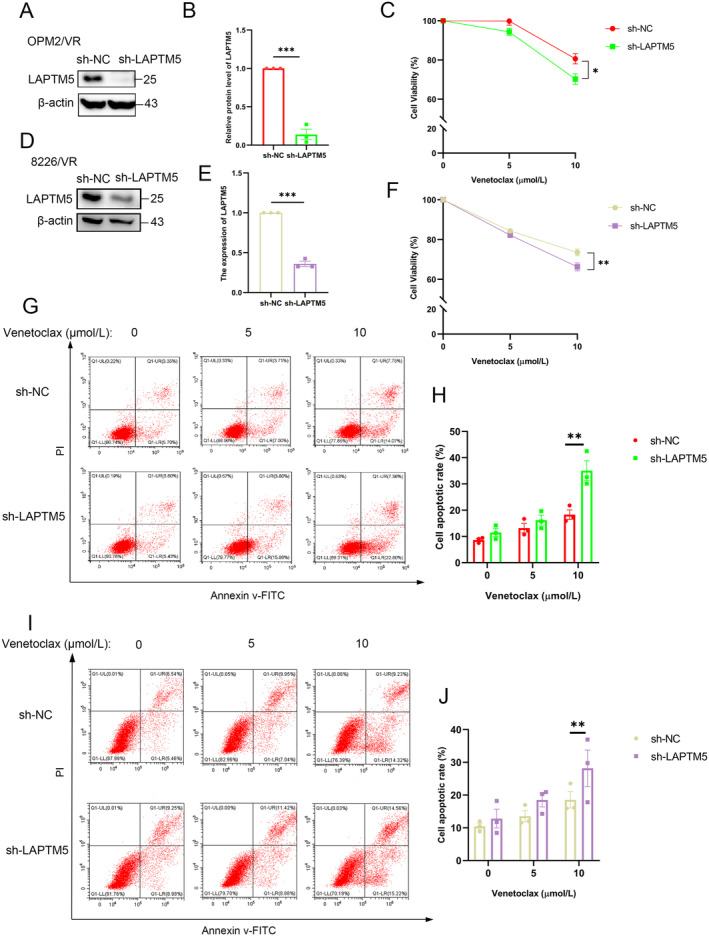
Depletion of LAPTM5 Sensitises Resistant Cells to Venetoclax. (A) Western blot analysis was employed to evaluate LAPTM5 expression levels in OPM2/VR(sh‐NC) and OPM2/VR(sh‐LAPTM5) cell lines. (B) LAPTM5 protein levels in OPM2/VR(sh‐NC) and OPM2/VR(sh‐LAPTM5) cells were quantified via Western blotting. Data represent the mean ± SEM from three independent experiments, normalised to the ‘sh‐NC’ group. (C) The impact of venetoclax treatment (0, 5, 10 μM) on the viability of OPM2/VR(sh‐NC) and OPM2/VR(sh‐LAPTM5) cells was assessed after 48 h using a CCK‐8 assay, presenting mean ± SEM. (D) Analysis of LAPTM5 expression in 8226/VR(sh‐NC) and 8226/VR(sh‐LAPTM5) cells was conducted using Western blotting. (E) LAPTM5 protein levels were quantified in 8226/VR(sh‐NC) and 8226/VR(sh‐LAPTM5) cells, with mean ± SEM values derived from three experiments, normalised to the ‘sh‐NC’ group. (F) The viability of 8226/VR(sh‐NC) and 8226/VR(sh‐LAPTM5) cells following 48‐h treatment with Venetoclax (0, 5, 10 μM) was measured using the CCK‐8 assay, with results presented as mean ± SEM. (G) Flow cytometric analysis was utilised to assess apoptosis in OPM2/VR(sh‐LAPTM5) and OPM2/VR(sh‐NC) cells under varying concentrations of Venetoclax. (H) Apoptosis in OPM2/VR‐sh‐NC and OPM2/VR‐sh‐LAPTM5 cells was evaluated after 48 h of Venetoclax treatment (0, 5, 10 μM) by Annexin V/ PI staining, presenting mean ± SEM results. (I) Flow cytometric assessment of apoptosis was also performed in 8226/VR(sh‐LAPTM5) and 8226/VR(sh‐NC) cells across different venetoclax concentrations. (J) Evaluation of apoptosis in 8226/VR‐sh‐NC and 8226/VR‐sh‐LAPTM5 cells post‐48‐h venetoclax treatment (0, 5, 10 μM) was conducted through Annexin V/PI staining, with results expressed as mean ± SEM. *p < 0.05, **p < 0.01, ***p < 0.001.

To delineate the function of LAPTM5 in venetoclax resistance, we utilised small hairpin RNA (sh‐LAPTM5) to stably knock down LAPTM5 expression in OPM2/VR cells (Figure 3A,B). Subsequent assessments of cell viability upon venetoclax treatment revealed that downregulation of LAPTM5 leads to reduced viability in OPM2/VR cells, particularly at a concentration of 10 μM venetoclax (Figure 3C). Similar results were obtained in 8226 cells, indicating the cellular effect is specific (Figure 3D–F). We next assess the cell apoptotic rate, interestingly, knocking down of LAPTM5 induces a higher rate of apoptotic cells upon the venetoclax treatment in both OPM2/VR cells (Figure 3G,H) and 8226/VR cells (Figure 3I,J). We next measured the cleaved caspase 3 and found knockdown of LAPTM5 enhanced cleaved caspase 3 level upon venetoclax treatment (Figure S2A–C). Similar data were documented in both OPM2/VR cells (Figure S2A–C) and 8226/VR cells (Figure S2D–F). These results indicate that LAPTM5 is required for developing resistance to venetoclax in MM, possibly by suppressing the cell apoptosis.

Since other LAPTM family members are involved in lysosomal processes, we next silenced another LAPTM member LAPTM4B in venetoclax‐resistant OPM2 (Figure S3A,B) and 8226 cell lines (Figure S3D,E) using small interfering RNA. Our CCK8 assays demonstrated that LAPTM4B knockdown did not alter sensitivity to venetoclax (Figure S3C,F), supporting the specificity of LAPTM5 in conferring resistance.

### 3.3 Overexpression of LAPTM5 Promotes Drug Resistance to Venetoclax in Multiple Myeloma

**FIGURE 4:**
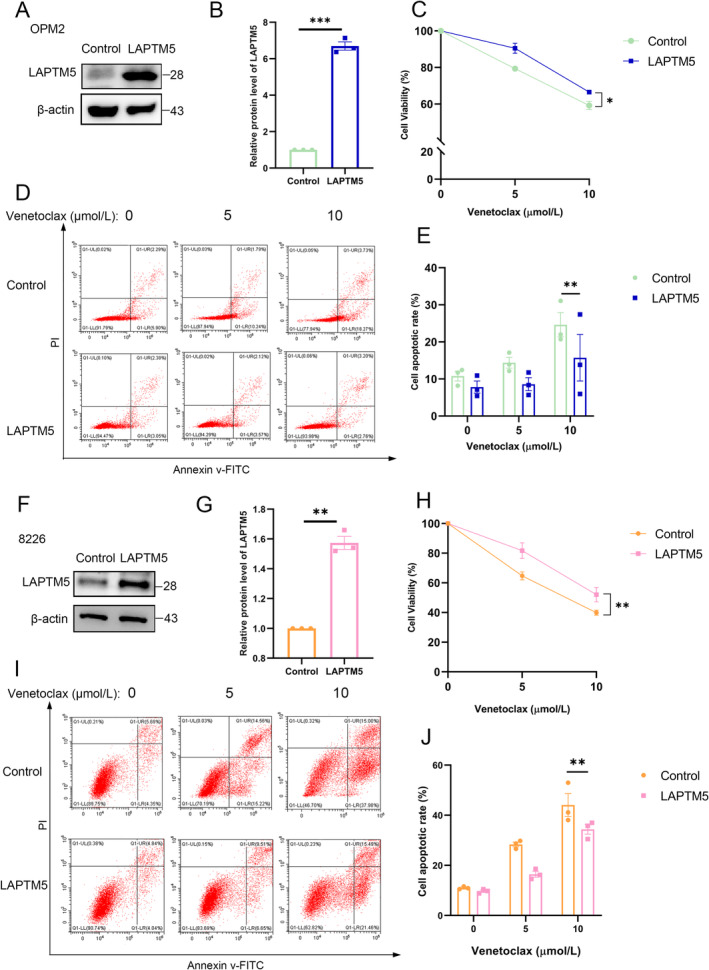
Overexpression of LAPTM5 Enhances Cellular Resistance to Venetoclax. (A) Western blotting was utilised to detect LAPTM5 expression levels in OPM2‐Control and OPM2‐LAPTM5 cells. (B) LAPTM5 protein levels were quantified, with mean ± SEM data from three independent experiments normalised to the ‘Control’ group; statistical significance was confirmed with a paired t‐test, yielding ***p < 0.001. (C) OPM2‐Control and OPM2‐LAPTM5 cells were subjected to venetoclax treatment (0, 5, 10 μM) for 48 h, and their viability was assessed through CCK‐8 analysis, reporting mean ± SEM. (D) Flow cytometric analysis was conducted to evaluate apoptosis in OPM2‐Control and OPM2‐LAPTM5 cells at various venetoclax concentrations. (E) Apoptosis in OPM2‐Control and OPM2‐LAPTM5 cells following treatment with venetoclax (0, 5, 10 μM) for 48 h was assessed via Annexin V/ PI staining, with results expressed as mean ± SEM. (F) LAPTM5 expression levels in 8226‐Control and 8226‐LAPTM5 cells were detected through Western blotting. (G) LAPTM5 protein levels were quantified, presenting mean ± SEM data from three experiments normalised to the ‘Control’ group. (H) The viability of 8226‐Control and 8226‐LAPTM5 cells post‐48‐h venetoclax treatment (0, 5, 10 μM) was measured using the CCK‐8 assay. (I) Flow cytometric detection of apoptosis in 8226‐Control and 8226‐LAPTM5 cells at indicated venetoclax concentrations was also conducted. (J) Apoptosis assessment in 8226‐Control and 8226‐LAPTM5 cells following 48‐h venetoclax treatment (0, 5, 10 μM) was performed using Annexin V/PI staining, presenting mean ± SEM. * *p* < 0.05, ** *p* < 0.01, *** *p* < 0.001.

To ascertain the molecular function of LAPTM5, we generated OPM2 cells stably expressing LAPTM5 (Figure 4A,B), which demonstrated increased resistance to venetoclax (Figure 4C). Next, we evaluated the apoptotic response. As anticipated, LAPTM5 overexpression significantly decreased the apoptotic percentage compared to the control cells, upon the treatment of various concentrations of venetoclax (Figure 4D,E). We next generated 8226 cells stably expressing LAPTM5 (Figure 4F,G), and observed that overexpression of LAPTM5 enhanced resistance to venetoclax (Figure 4H), as well as decreased cellular apoptotic rate (Figure 4I,J). Furthermore, the cleaved caspase 3 levels were reduced in stably LAPTM5 expressing OPM2 and 8826 cells, compared to the control cells (Figure S4A–F). These data together demonstrate that upregulation of LAPTM5 enhances the development of venetoclax resistance in MM.

Given that venetoclax is a well‐established Bcl‐2 inhibitor, we thereafter performed western blot analysis of Bcl‐2 protein levels in parental cells, venetoclax‐resistant cells and MM cells with LAPTM5 overexpression. Our data showed that Bcl‐2 level was increased in resistant cells, compared to the parental cells (Figure S5A,B). Moreover, LAPTM5 overexpression increased Bcl‐2 levels in both OPM2 and 8226 cells (Figure S5C,D). These findings suggest that LAPTM5‐mediated venetoclax resistance could be Bcl‐2 dependent. Additionally, we treated MM cell lines with alternative Bcl‐2 inhibitors, ABT‐737 and Navitoclax, and performed CCK8 assays. The results demonstrate that LAPTM5 overexpression reduces sensitivity to these inhibitors (Figure S5E,F). Conversely, knockdown of LAPTM5 enhances sensitivity (Figure S5G,H). These findings suggest that LAPTM5's role in drug resistance may extend to other Bcl‐2‐targeted therapies.

### 3.4 LAPTM5 Facilitates the Fusion of Autophagosomes With Lysosomes in Multiple Myeloma

**FIGURE 5:**
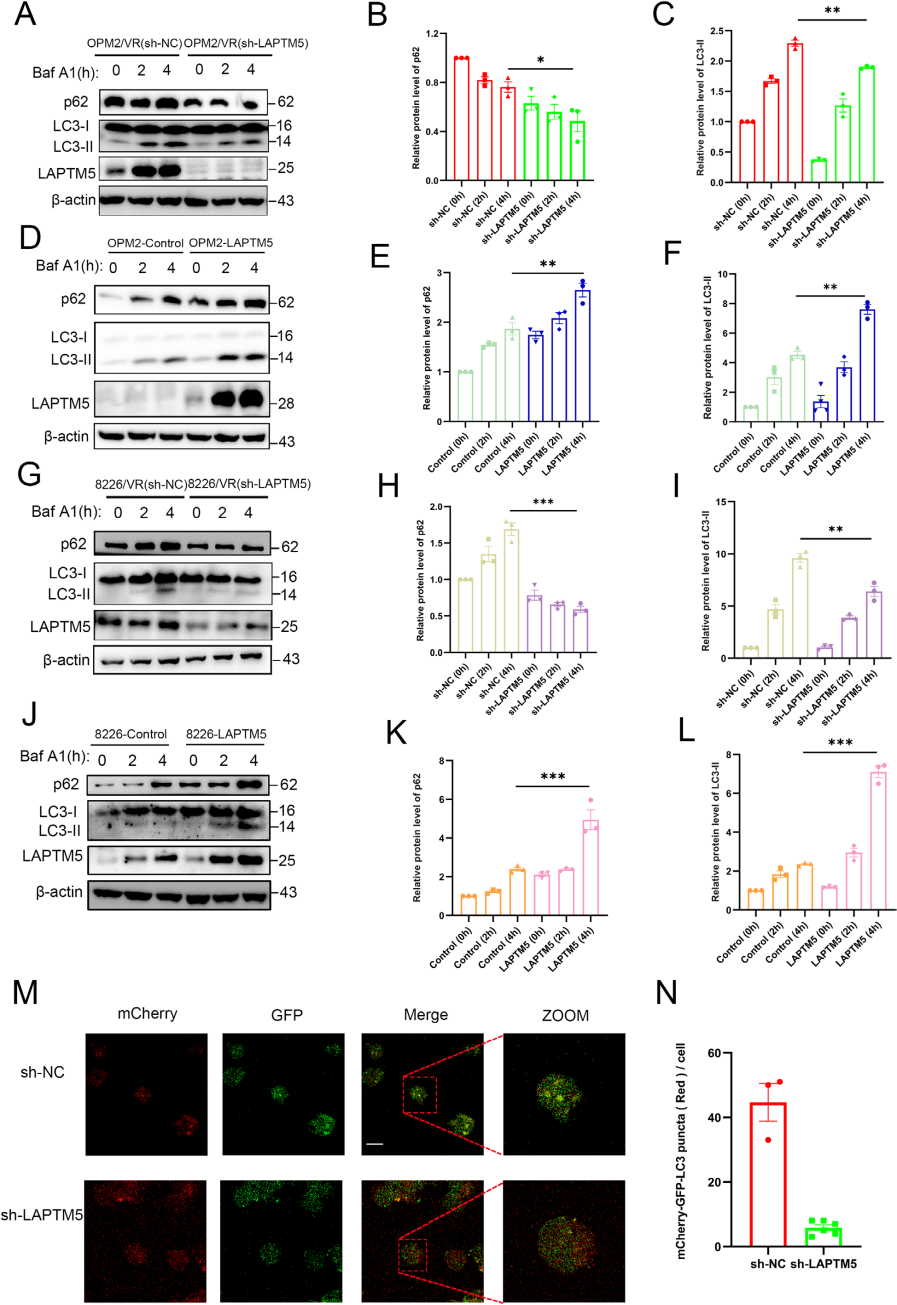
LAPTM5 Facilitates Autophagosome‐Lysosome Fusion. (A) The expression levels of p62 and LC3‐II were assessed via Western blotting in OPM2/VR cells following LAPTM5 knockdown. (B) p62 protein levels were quantified in OPM2/VR‐sh‐NC and OPM2/VR‐sh‐LAPTM5 cells treated with 1 μM BafA1 for durations of 0, 2 and 4 h. Data represent mean ± SEM from three independent experiments, normalised to the ‘sh‐NC’ group. (C) LC3‐II protein levels were similarly quantified in OPM2/VR‐sh‐NC and OPM2/VR‐sh‐LAPTM5 cells treated with 1 μM BafA1 for 0, 2 and 4 h. (D) Western blot analysis was conducted to evaluate p62 and LC3‐II expression in OPM2 cells overexpressing LAPTM5. (E) p62 protein levels in OPM2‐Control and OPM2‐LAPTM5 cells were quantified after treatment with 1 μM BafA1 for 0, 2 and 4 h. The data represented as mean ± SEM from three experiments. (F) LC3‐II protein levels were assessed in OPM2‐Control and OPM2‐LAPTM5 cells, following treatment with 1 μM BafA1 for the aforementioned time points. Mean ± SEM data were obtained from three experiments. (G) The expression levels of p62 and LC3‐II were also analysed in 8226/VR cells subjected to LAPTM5 knockdown via Western blotting. (H) p62 protein levels in 8226/VR‐sh‐NC and 8226/VR‐sh‐LAPTM5 cells treated with 1 μM BafA1 for 0, 2 and 4 h were quantified, with results presented as mean ± SEM. (I) LC3‐II protein levels were analysed in 8226/VR‐sh‐NC and 8226/VR‐sh‐LAPTM5 cells following BafA1 treatment. (J) Western blot analysis of p62 and LC3‐II expression was performed in 8226 cells overexpressing LAPTM5. (K) p62 protein levels in 8226‐Control and 8226‐LAPTM5 cells were assessed after treatment with 1 μM BafA1 for 0, 2 and 4 h. (L) LC3‐II protein levels were similarly analysed in 8226‐Control and 8226‐LAPTM5 cells after BafA1 treatment. (M) Autophagic activity was visualised under a confocal microscope in OPM2/VR(sh‐NC) and OPM2/VR(sh‐LAPTM5) cells infected with mCherry‐GFP‐LC3 virus 48 h post 1 μM BafA1 treatment. Scale bar: 20 μm. (N) Quantitative analysis of mCherry‐GFP‐LC3 expression following 48‐h infection with the mCherry‐GFP‐LC3 virus and subsequent addition of 1 μM BafA1 demonstrated statistical significance. * *p* < 0.05, ** *p* < 0.01, ***p < 0.001.

Given that autophagy is a central process regulating drug resistance [15, 39, 40], we hypothesise that LAPTM5 enhances venetoclax resistance in MM by modulating autophagic activity. To test this hypothesis, we first evaluated autophagy levels in OPM2/VR cells with and without the depletion of LAPTM5. We observed a reduction in LC3‐II and p62 levels in OPM2/VR cells after LAPTM5 depletion upon the treatment of Bafilomycin A1 (BafA1, 1 μM) over time (0, 2 and 4 h) (Figure 5A–C). Conversely, overexpression of LAPTM5 in OPM2 cells resulted in elevated LC3‐II and p62 levels following BafA1 treatment compared to control cells (Figure 5D–F). Similar results were observed in both OPM2 (Figure 5A–F) and 8226 cells (Figure 5G–L). Additionally, we measured ATG5 expression and found that LAPTM5 depletion or overexpression does not significantly alter ATG5 levels (Figure S6A–D), suggesting its specific role in autophagosome‐lysosome fusion, rather than autophagy initiation.

To further investigate autophagosome formation, we employed a fluorescence assay utilising mCherry‐GFP‐LC3, where the GFP signal is sensitive to acidic conditions while mCherry remains stable. Our results demonstrated a markedly drop of mCherry‐GFP‐LC3 puncta (red) per cell in LAPTM5‐depleted cells (Figure 5M,N), suggesting that LAPTM5 promotes the fusion of autophagosomes with lysosomes.

These data from LC3 quantification and fluorescence assays provide compelling evidence that LAPTM5 enhances autophagy by facilitating the fusion of autophagosomes with lysosomes.

### 3.5 Autophagy Is Required for LAPTM5 Promotion of Venetoclax Resistance in MM

**FIGURE 6:**
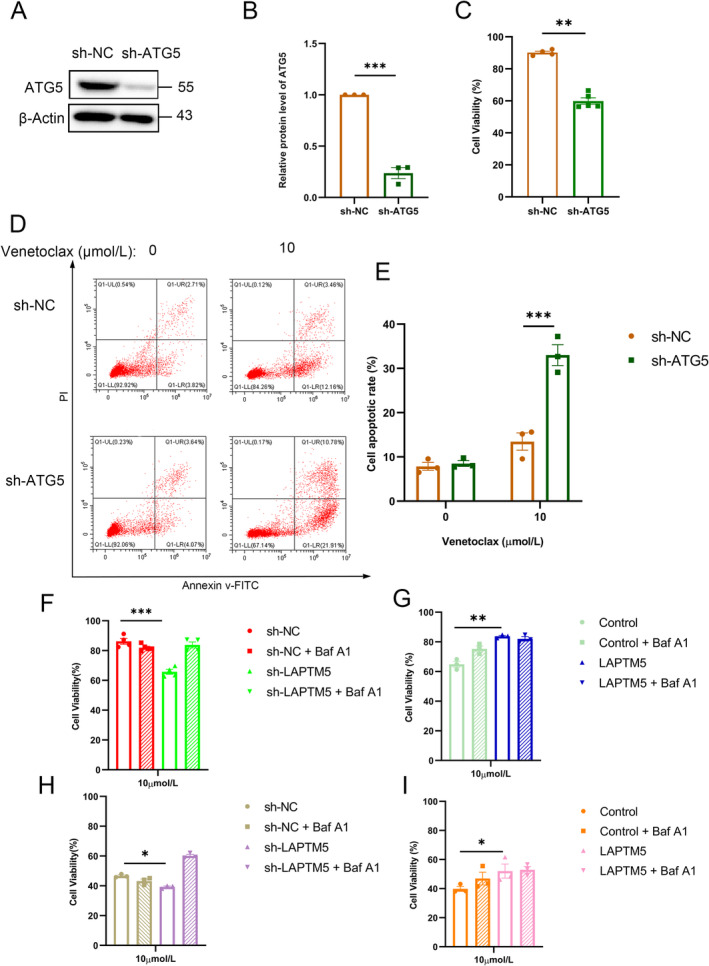
Autophagy is Essential for LAPTM5‐Mediated Venetoclax Resistance in Multiple Myeloma. (A) Western blot analysis was employed to assess ATG5 protein expression levels in OPM2/VR (sh‐NC) and OPM2/VR (sh‐ATG5) cells. (B) The quantification of ATG5 protein levels was performed via Western blotting, with mean ± SEM reported from three independent experiments. (C) OPM2/VR(sh‐NC) and OPM2/VR(sh‐ATG5) cells were treated with venetoclax (0, 10 μM) for 48 h, after which cell viability was assessed using CCK‐8, with data presented as mean ± SEM. (D) Apoptotic rates were evaluated using flow cytometry in OPM2/VR (sh‐NC) and OPM2/VR (sh‐ATG5) cells. (E) Following venetoclax treatment (0, 10 μM) for 48 h, apoptosis in OPM2/VR(sh‐NC) and OPM2/VR(sh‐ATG5) cells was assessed via Annexin V/PI staining, with statistical analysis performed using paired *t*‐test. (F) OPM2/VR(sh‐NC) and OPM2/VR(sh‐LAPTM5) cells were treated with Venetoclax (0, 10 μM) and 10 nM BafA1 for 48 h; thereafter, cell viability was determined via CCK‐8, with mean ± SEM. (G) OPM2‐Control and OPM2‐LAPTM5 cells subjected to venetoclax (0, 10 μM) and 10 nM BafA1 treatment for 48 h showed cell viability results evaluated by CCK‐8, presenting mean ± SEM. (H) 8226/VR(sh‐NC)and 8226/VR(sh‐LAPTM5) cells were treated with venetoclax (0, 10 μM) and 10 nM BafA1 for 48 h, then cell viability was determined by CCK‐8 mean ± SEM, and results are obtained using paired *T*‐test, *p < 0.05. (I) 8226‐Control and 8226‐LAPTM5 cells were treated with venetoclax (0, 10 μM) and 10 nM BafA1 for 48 h, then cell viability was determined by CCK‐8 mean ± SEM, and results are obtained using paired *T*‐test, *p < 0.05, **p < 0.01, ***p < 0.001.

To further establish that autophagy mediates the pro‐resistance effects of LAPTM5 in MM, we employed a knockdown strategy targeting ATG5 (sh‐ATG5) in OPM2/VR cells (Figure 6A,B), as ATG5 is a pivotal regulator in autophagy initiation [41]. Our assessment of cell viability indicated that depletion of ATG5 resulted in decreased cell viability in OPM2/VR cells treated with venetoclax (Figure 6C). Moreover, we evaluated the effects of venetoclax on apoptosis and observed increased apoptotic levels in ATG5 knockdown cells compared to controls (Figure 6D,E). Subsequently, we utilised Bafilomycin A1 (BafA1) to inhibit the fusion of autophagosomes with lysosomes and found that BafA1 treatment abrogated the loss of cell viability induced by LAPTM5 shRNA during venetoclax treatment (Figure 6F), as well as diminished the enhanced drug resistance induced by LAPTM5 overexpression (Figure 6G). Similar phenotypes were noted in both OPM2 and 8226 cells (Figure 6H,I), indicating that autophagic initiation, as well as the fusion of autophagosomes with lysosomes, are essential for LAPTM5 promoting venetoclax resistance.

### 3.6 Nutritional Deficiencies‐Induced Autophagy Enhances LAPTM5 Expression

**FIGURE 7:**
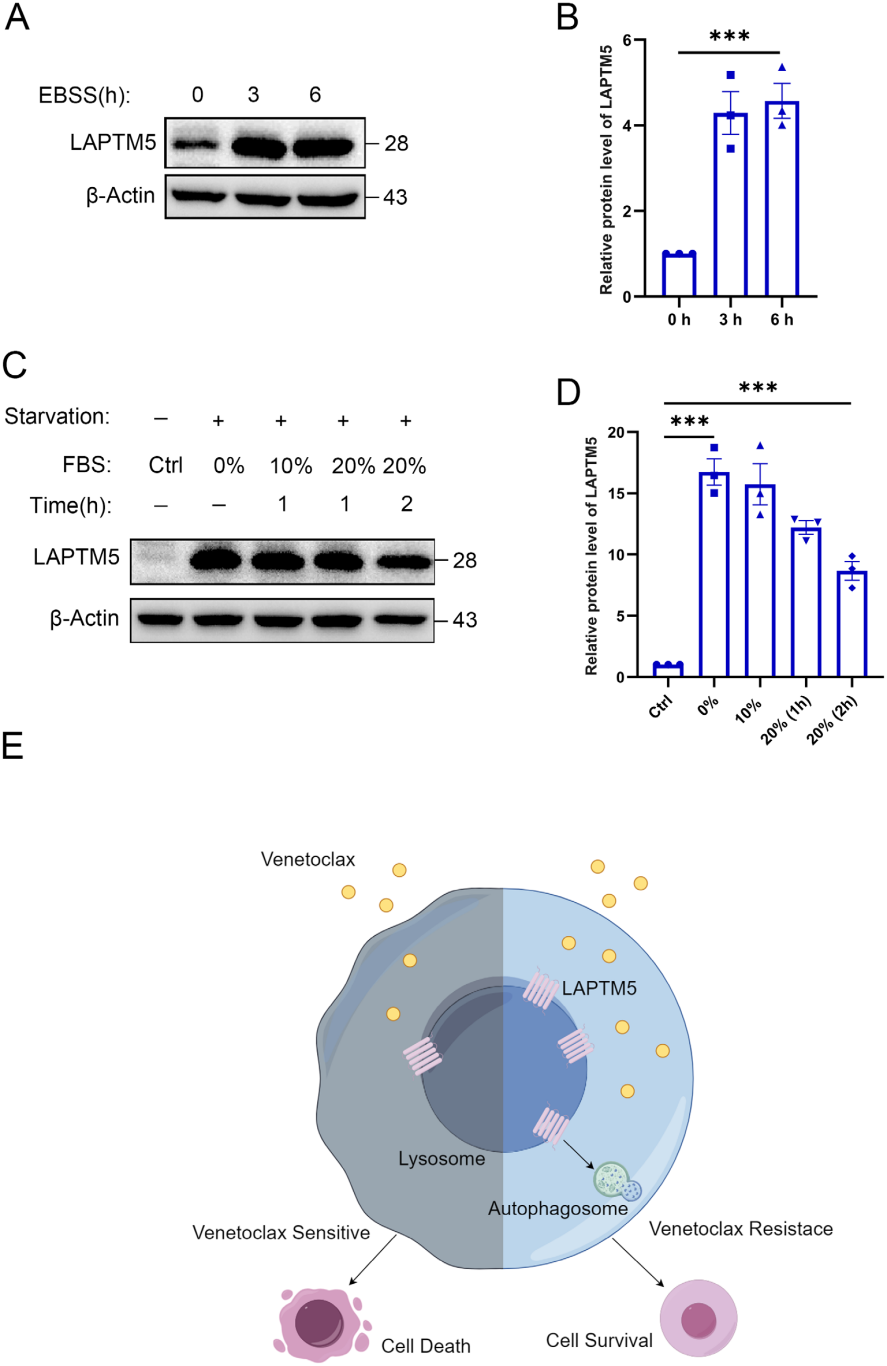
Nutritional Deficiency Induces Upregulation of LAPTM5. (A) The levels of LAPTM5 protein were assessed by Western blot analysis in OPM2 cells subjected to treatment with EBSS for durations of 0, 3 and 6 h. (B) Quantitative analysis of the data from three independent experiments in (A) is presented as mean ± SEM, with values normalised to the ‘Control’ group. (C) OPM2 cells were cultured in serum‐free medium for 12 h, followed by exposure to media containing 10% or 20% FBS for 1 or 2 h. Western blotting was employed to quantify LAPTM5 expression levels. (D) Statistical analysis of the Western blot results from (C) was conducted, with significance assessed using paired *t*‐tests (***p < 0.001). (E) A schematic representation illustrating the conceptual framework and primary findings of this study is provided. It highlights the upregulation of LAPTM5 in multiple myeloma (MM) resistant cells, which enhances cell proliferation and promotes apoptotic resistance through the facilitation of autophagy. Consequently, LAPTM5 is implicated in conferring resistance to venetoclax in MM.

Lysosomes serve as the primary organelles for nutrient sensing within the cell [42], while autophagy is intricately regulated by cellular nutrient availability [43]. Despite the significance of LAPTM5 as a critical lysosomal membrane protein, its role in nutrient sensing and the autophagic responses to nutritional deficiencies remains largely unexplored. Herein, we questioned whether the nutritional status of MM cells regulates LAPTM5 expression. OPM2 cells were subjected to Earle's Balanced Salt Solution (EBSS) treatment for durations of 0, 3 and 6 h. Western blot analysis revealed a dose‐dependent increase in LAPTM5 protein levels during EBSS treatment (Figure 7A,B). To further elucidate the relationship between LAPTM5 expression and nutrient sensing, we reintroduced serum into the medium following cellular starvation. As anticipated, LAPTM5 levels were upregulated during starvation, with serum refeeding leading to a marked reduction in LAPTM5 expression (Figure 7C,D). These findings collectively indicate that LAPTM5 expression rapidly responses to cellular nutritional state. To further investigate whether this nutrient response is directly linked to venetoclax resistance, we assessed drug resistance under normal nutrient conditions as well as under serum starvation. Our data, derived from LAPTM5‐depleted venetoclax‐resistant OPM2 cells and LAPTM5‐stably expressing OPM2 cells (Figure S7A,B), showed that LAPTM5‐mediated venetoclax resistance is regulated by nutrient availability.

In summary, our study highlighted the function of upregulated LAPTM5 in conferring venetoclax resistance in MM. We demonstrate that LAPTM5 facilitates autophagosome‐lysosome fusion, thereby promoting drug resistance in malignant cells, underscoring the potential role of LAPTM5 in overcoming drug resistance in MM (Figure 7E).

## 4 Discussion

The incidence of multiple myeloma has been rising consistently in recent years. Despite ongoing efforts to develop effective treatments, the prognosis for patients remains discouraging, characterised by short median survival rates and a high propensity for relapse [44]. Drug resistance presents a complex and inevitable challenge that poses significant threats to patient health. Therefore, identifying therapeutic targets to combat drug resistance and elucidating their mechanisms in tumour biology is crucial for advancing cancer diagnosis and treatment.

Venetoclax, a key agent in the treatment of haematological malignancies, shows promise for multiple myeloma therapy [45]. However, resistance to venetoclax frequently develops, severely impacting patient outcomes [46]. The fundamental nature of tumour resistance involves the evasion of drug‐induced apoptosis [47], positioning autophagy and apoptosis as central themes in our investigation of resistance mechanisms. Within the autophagic process, lysosomes serve as critical mediators, as they are essential for the intracellular degradation that characterises autophagy [48].

In this study, we observed that LAPTM5 expression is significantly elevated in patients with relapsed multiple myeloma, as indicated by data from the COMMPASS database. Moreover, the venetoclax resistant OPM2 cells (OPM2/VR) and 8226 cells (8226/VR) exhibited higher LAPTM5 levels compared to the parental cells. We thereafter performed a series of cellular experiments to investigate the functions of LAPTM5 for drug resistance in multiple myeloma and its associated molecular pathways. Our findings indicate LAPTM5 confers resistance to venetoclax. Functional assays demonstrated that silencing LAPTM5 reduced cell viability and enhanced apoptosis in both OPM2/VR cells and 8226/VR cells upon the venetoclax treatment. Collectively, these results suggest that the overexpression of LAPTM5 promotes the venetoclax resistance, possibly via suppressing the apoptosis of myeloma cells.

Autophagy is a central biological process in drug resistance [15] and our data confirmed that LAPTM5 promotes autophagy. Additionally, we established ATG5‐knockdown cell lines and evaluated the proliferation and apoptosis of these cells in response to venetoclax treatment, revealing a diminished sensitivity to the drug in ATG5‐depleted cells. This underscores the relationship between autophagy and drug resistance, highlighting that the inhibition of autophagy can mitigate resistance.

To further investigate the role of LAPTM5‐regulated autophagy in resistance to multiple myeloma, we administered varying doses of venetoclax to both LAPTM5‐knockdown and control cells. Our results indicated that BafA1 treatment reduced the sensitivity of LAPTM5‐expressing cells to the drug. Therefore, we anticipate that the role of LAPTM5 in promoting drug resistance is contingent upon its regulatory effects on autophagy.

In conclusion, this study elucidates the function of LAPTM5 on venetoclax resistance in multiple myeloma through its regulation of autophagy. Nevertheless, further investigations, including proteomic analyses such as mass spectrometry, are needed to identify LAPTM5‐associated proteins and to gain deeper insights into its influence on autophagy and multiple myeloma.

While our study focuses on the relationship between LAPTM5 and drug resistance in multiple myeloma, it is noteworthy that our exploration of the promoting effect of LAPTM5 on autophagy revealed a positive correlation between autophagy enhancement and increased LAPTM5 protein expression. This observation fills an existing gap in the literature regarding LAPTM5 in drug resistance. LAPTM5 may play a critical role in various haematological malignancies and solid tumours, suggesting that its investigation warrants further attention.

## Author Contributions

**Yuxiang Li:** conceptualization (equal), data curation (equal), investigation (equal), methodology (equal), resources (equal), software (equal). **Jing Bai:** conceptualization (equal), methodology (equal), resources (equal), software (equal), validation (equal). **Dan Liu:** validation (equal). **Jinxia Hao:** software (equal). **Ruyu Yan:** investigation (equal). **Hongjuan Guo:** investigation (equal). **Yuzhi Huang:** investigation (equal). **Hongtao Yu:** investigation (equal). **Hao Leng:** investigation (equal). **Kecheng Zhou:** funding acquisition (equal), supervision (equal), visualization (equal), writing – review and editing (equal). **Minxia Liu:** funding acquisition (equal), supervision (equal).

## Conflicts of Interest

The authors declare no conflicts of interest.

## Supporting information


**Figure S1.** Differential Expression of Lysosomal Membrane Protein Genes Between Normal and Multiple Myeloma (MM) Samples.
**Figure S2.** Knockdown of LAPTM5 Protein Increases Sensitivity and Promotes Apoptosis of Drug‐Resistant Cells in Response to Venetoclax.
**Figure S3.** Knockdown of LAPTM4B Does Not Alter Cell Sensitivity to Venetoclax.
**Figure S4.** LAPTM5 Inhibits Apoptosis Levels Upon the Venetoclax Treatment.
**Figure S5.** LAPTM5‐Mediated Venetoclax Resistant Is Dependent on Bcl‐2.
**Figure S6.** LAPTM5 Does Not Regulate ATG5 expression.
**Figure S7.** LAPTM5‐mediated Venetoclax Resistance is Regulated by Nutrient Availability.

## Data Availability

All the data generated during the current study are available from the corresponding author on reasonable request.
