# Supplementary material for: LAPTM5 Confers the Resistance to Venetoclax via Promoting the Autophagosome‐Lysosome Fusion in Multiple Myeloma
Source: J Cell Mol Med. 2025 Jan 3;29(1):e70331. doi: 10.1111/jcmm.70331 (PMC11702483; doi:10.1111/jcmm.70331)
Supplement: Supplementary file 1 — Figure S1. Differential Expression of Lysosomal Membrane Protein Genes Between Normal and Multiple Myeloma (MM) Samples. Figure S2. Knockdown of LAPTM5 Protein Increases Sensitivity and Promotes Apoptosis of Drug‐Resistant Cells in Response to Venetoclax. Figure S3. Knockdown of LAPTM4B Does Not Alter Cell Sensitivity to Venetoclax. Figure S4. LAPTM5 Inhibits Apoptosis Levels Upon the Venetoclax Treatment. Figure S5. LAPTM5‐Mediated Venetoclax Resistant Is Dependent on Bcl‐2. Figure S6. LAPTM5 Does Not Regulate ATG5 expression. Figure S7. LAPTM5‐mediated Venetoclax Resistance is Regulated by Nutrient Availability. [file JCMM-29-e70331-s001.pdf]

LAPTM5 Confers the Resistance to Venetoclax via Promoting the Autophagosome-Lysosome Fusion in Multiple Myeloma

Yuxiang Li[1,#], Jing Bai[1,#], Dan Liu[1,2], Jinxia Hao[3], Ruyu Yan[1,2] ,Hongjuan Guo[1],Yuzhi Huang[1],Hongtao Yu[1], Hao Leng[1], Kecheng Zhou[1,2*], Minxia Liu[1*]

[1] School of Life Sciences, Anhui Medical University, Hefei, 230032, China

[2] Department of Biochemistry and Molecular Biology, School of Basic Medical Sciences, Anhui Medical University, Hefei, 230032, China

[3] Department of Internal Medicine, Xi'an Jiaotong University Hospital, Xi'an, 710049, China

[#] Yuxiang Li and Jing Bai contribute equally to the current study

Supplementary Information

A

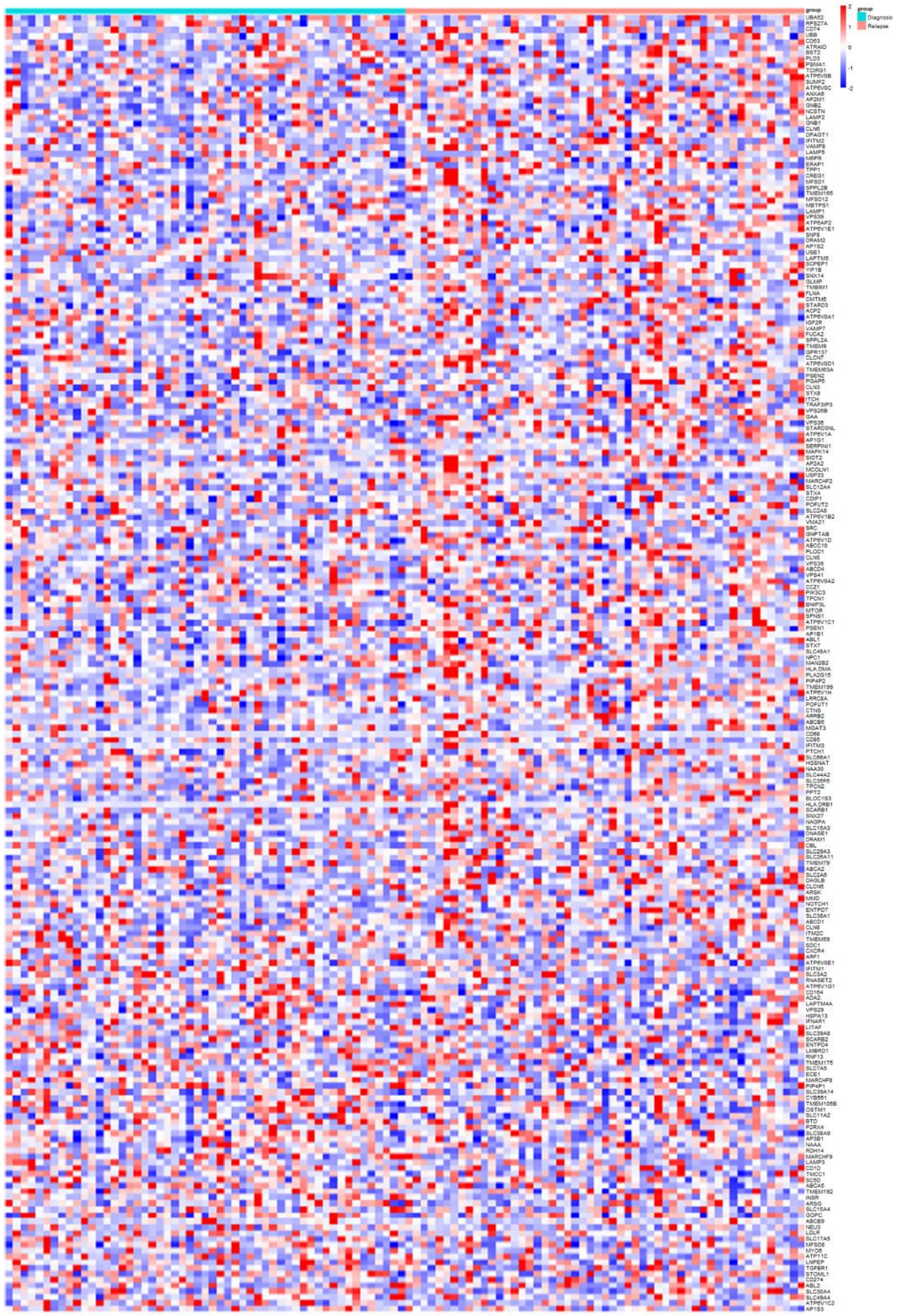

B

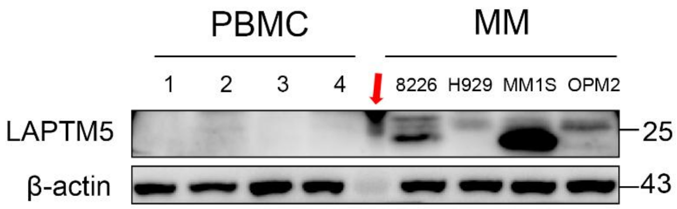

Supplementary Figure S1. Differential Expression of Lysosomal Membrane Protein Genes Between Normal and Multiple Myeloma (MM) Samples

(A) Heatmap illustrating the differential expression of lysosomal membrane protein genes between diagnostic and relapsed MM patients.
(B) Western blot analysis showing LAPTM5 protein expression levels in MM cell lines, compared with Peripheral blood mononuclear cells (PBMCs). Red arrows indicate the band from protein marker on the Western blot.

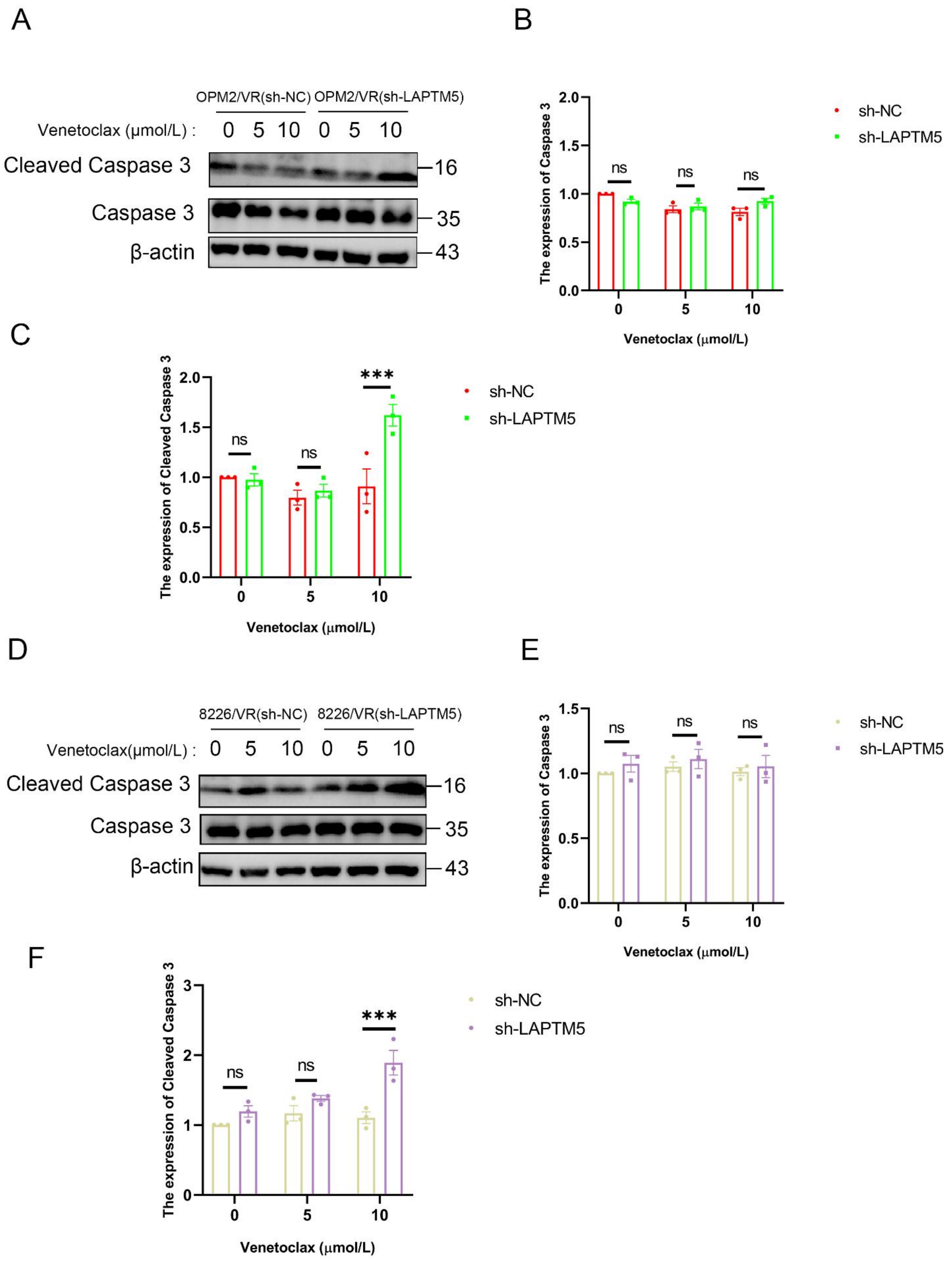
A
OPM2/VR(sh-NC)
OPM2/VR(sh-LAPTM5)
Venetoclax (μmol/L) :
0 5 10 0 5 10
Cleaved Caspase 3
16
Caspase 3
35
β-actin
43
B
The expression of Caspase 3
2.0
1.5
1.0
0.5
0.0
ns
ns
ns
0
5
10
Venetoclax (μmol/L)
sh-NC
sh-LAPTM5
C
The expression of Cleaved Caspase 3
2.0
1.5
1.0
0.5
0.0
ns
ns
***
0
5
10
Venetoclax (μmol/L)
sh-NC
sh-LAPTM5
D
8226/VR(sh-NC)
8226/VR(sh-LAPTM5)
Venetoclax(μmol/L) :
0 5 10 0 5 10
Cleaved Caspase 3
16
Caspase 3
35
β-actin
43
E
The expression of Caspase 3
1.5
1.0
0.5
0.0
ns
ns
ns
0
5
10
Venetoclax (μmol/L)
sh-NC
sh-LAPTM5
F
The expression of Cleaved Caspase 3
3
2
1
0
ns
ns
***
0
5
10
Venetoclax (μmol/L)
sh-NC
sh-LAPTM5

Supplementary Figure S2. Knockdown of LAPTM5 Protein Increases Sensitivity and Promotes Apoptosis of Drug-Resistant Cells in Response to Venetoclax

(A) Western blot analysis of Caspase-3 and Cleaved Caspase-3 protein expression levels in OPM2/VR-sh-NC and OPM2/VR-sh-LAPTM5 cells treated with venetoclax (0 μM, 5 μM, 10 μM) for 48 hours.
(B) Quantitative analysis of Caspase-3 protein levels normalized to the sh-NC group. Statistical significance was assessed using paired t-tests.
(C) Quantitative analysis of Cleaved Caspase-3 protein levels normalized to the sh-NC group, with significance evaluated using paired t-tests (***$p < 0.001$).
(D) Western blot detection of Caspase-3 and Cleaved Caspase-3 proteins in 8226/VR-shNC and 8226/VR-sh-LAPTM5 cells following venetoclax treatment (0 μM, 5 μM, 10 μM) for 48 hours.
(E) Quantitative analysis of Caspase-3 protein levels normalized to the sh-NC group, with significance assessed using paired t-tests.
(F) Quantitative analysis of Cleaved Caspase-3 protein levels normalized to the sh-NC group, with significance evaluated using paired t-tests (***$p < 0.001$).

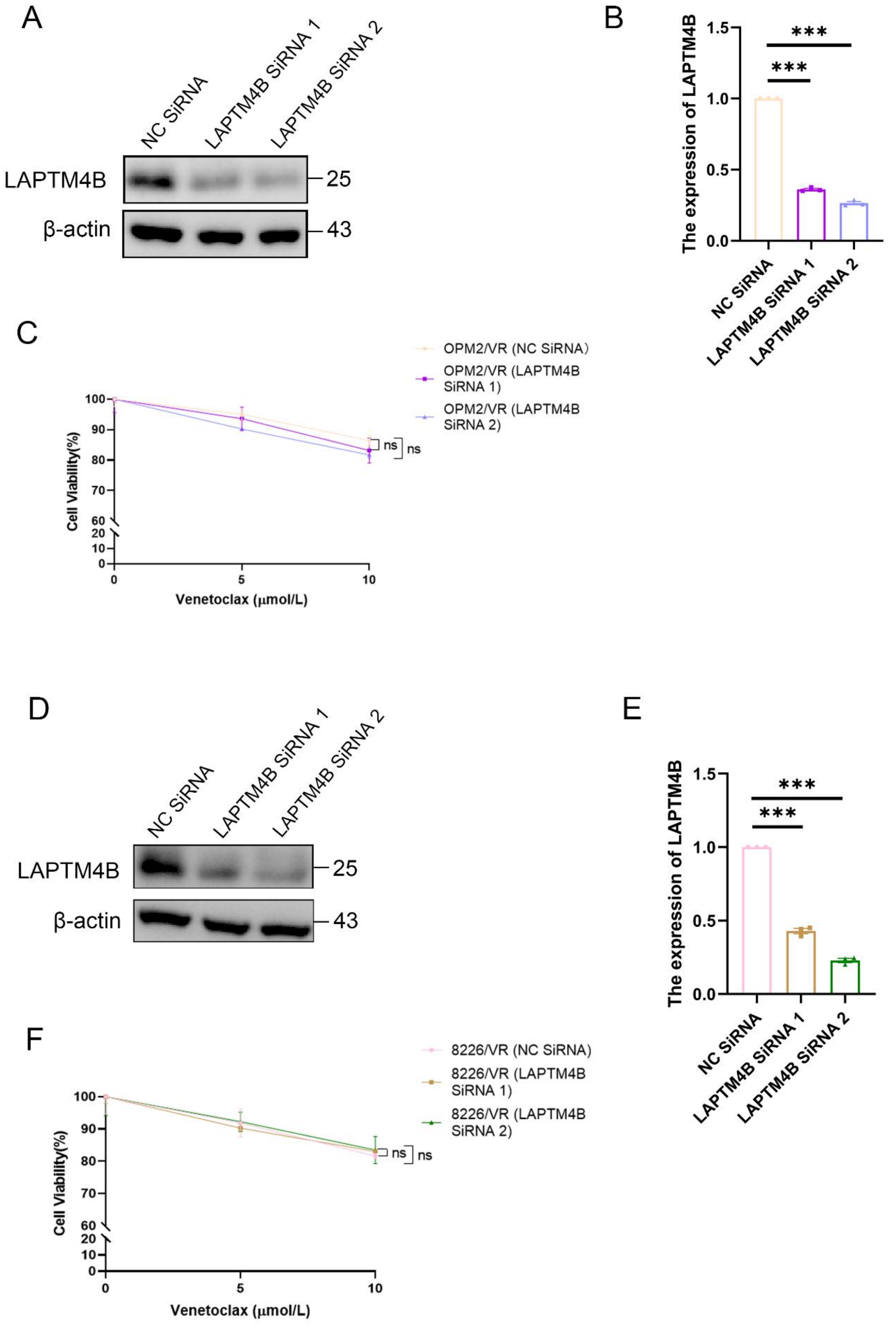

A
NC SiRNA
LAPTM4B SiRNA 1
LAPTM4B SiRNA 2
LAPTM4B
25
β-actin
43
B
The expression of LAPTM4B
1.5
1.0
0.5
0.0
***
***
NC SiRNA
LAPTM4B SiRNA 1
LAPTM4B SiRNA 2
C
OPM2/VR (NC SiRNA)
OPM2/VR (LAPTM4B SiRNA 1)
OPM2/VR (LAPTM4B SiRNA 2)
Cell Viability(%)
100
90
80
70
60
20
10
0
ns
ns
0
5
10
Venetoclax (μmol/L)
D
NC SiRNA
LAPTM4B SiRNA 1
LAPTM4B SiRNA 2
LAPTM4B
25
β-actin
43
E
The expression of LAPTM4B
1.5
1.0
0.5
0.0
***
***
NC SiRNA
LAPTM4B SiRNA 1
LAPTM4B SiRNA 2
F
8226/VR (NC SiRNA)
8226/VR (LAPTM4B SiRNA 1)
8226/VR (LAPTM4B SiRNA 2)
Cell Viability(%)
100
90
80
70
60
20
10
0
ns
ns
0
5
10
Venetoclax (μmol/L)

Supplementary Figure S3. Knockdown of LAPTM4B Does Not Alter Cell Sensitivity to Venetoclax

(A) Western blot analysis of LAPTM4B protein expression following knockdown in the OPM2/VR cells.

(B) Quantitative analysis of LAPTM4B levels in OPM2/VR cells transfected by siRNAs. Statistical significance was assessed using paired t-tests.

(C) Cell viability of OPM2/VR (SiNC), OPM2/VR (LAPTM4B SiRNA-1), and OPM2/VR (LAPTM4B SiRNA-2) cells treated with venetoclax for 48 hours was assessed using the CCK-8 assay. Data are presented as mean ± SEM.

(D) Western blot analysis of LAPTM4B protein expression following knockdown in the 8226/VR cells.

(E) Quantitative analysis of LAPTM4B levels in 8226/VR cells transfected by siRNAs. Statistical significance was assessed using paired t-tests.

(F) Cell viability of 8226/VR (SiNC), 8226/VR (LAPTM4B SiRNA-1), and 8226/VR (LAPTM4B SiRNA-2) cells treated with venetoclax for 48 hours, determined via CCK-8 assay. Data are presented as mean ± SEM.

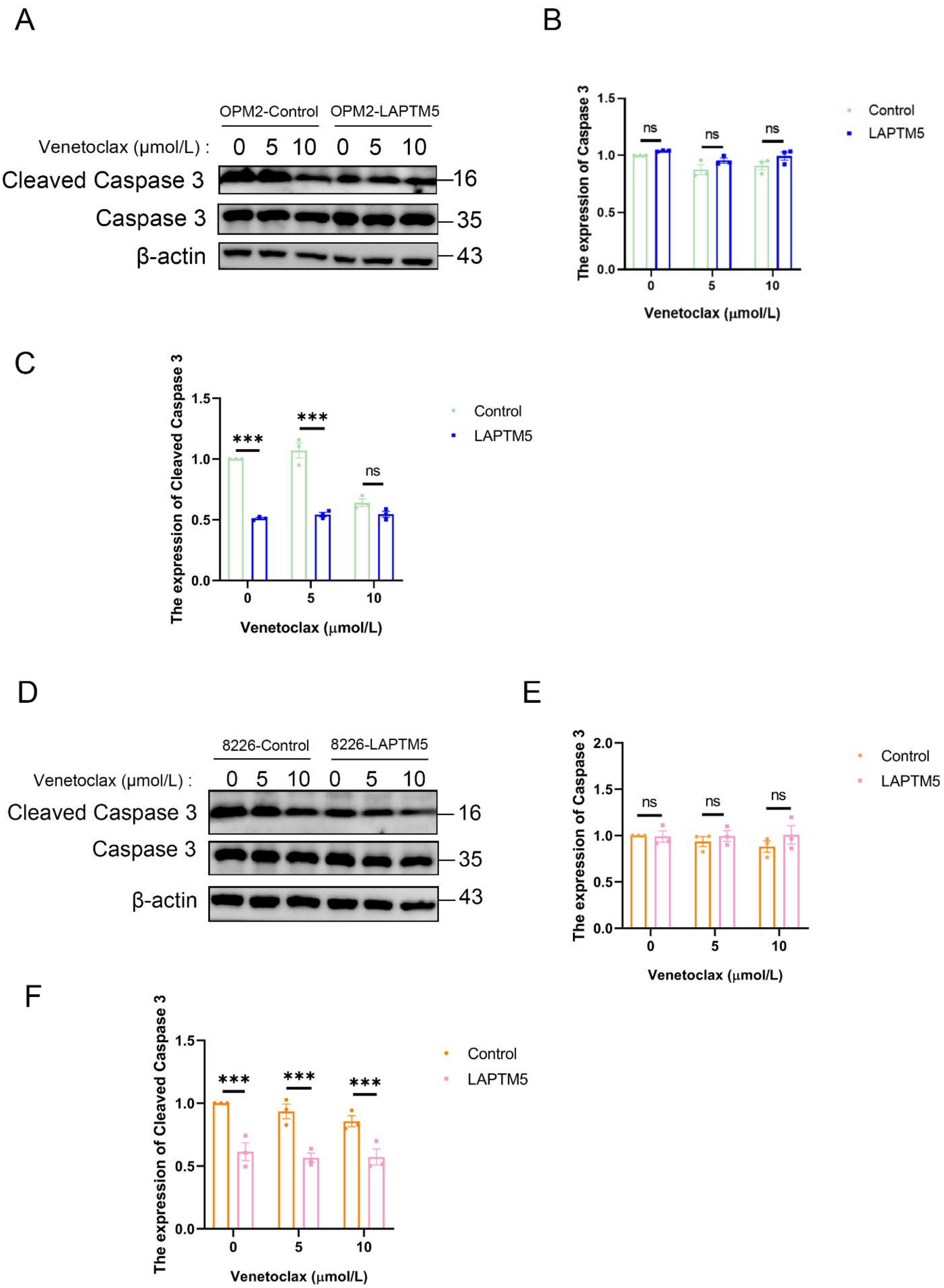
A
OPM2-Control
OPM2-LAPTM5
Venetoclax (μmol/L) :
0 5 10 0 5 10
Cleaved Caspase 3
Caspase 3
β-actin
16
35
43
B
The expression of Caspase 3
Venetoclax (μmol/L)
ns
Control
LAPTM5
C
The expression of Cleaved Caspase 3
Venetoclax (μmol/L)
***
ns
Control
LAPTM5
D
8226-Control
8226-LAPTM5
Venetoclax (μmol/L) :
0 5 10 0 5 10
Cleaved Caspase 3
Caspase 3
β-actin
16
35
43
E
The expression of Caspase 3
Venetoclax (μmol/L)
ns
Control
LAPTM5
F
The expression of Cleaved Caspase 3
Venetoclax (μmol/L)
***
Control
LAPTM5

Supplementary Figure S4. LAPTM5 Inhibits Apoptosis levels upon the Venetoclax treatment

(A) Western blot detection of Caspase-3 and Cleaved Caspase-3 protein expression levels in OPM2-Control and OPM2-LAPTM5 cells treated with venetoclax (0 μM, 5 μM, 10 μM) for 48 hours.

(B) Quantitative analysis of Caspase-3 protein levels normalized to the Control group, with significance assessed using paired t-tests.

(C) Quantitative analysis of Cleaved Caspase-3 protein levels normalized to the Control group, with significance evaluated using paired t-tests (***$p < 0.001$).

(D) Western blot detection of Caspase-3 and Cleaved Caspase-3 proteins in 8226-Control and 8226-LAPTM5 cells treated with venetoclax (0 μM, 5 μM, 10 μM) for 48 hours.

(E) Quantitative analysis of Caspase-3 protein levels normalized to the Control group, with significance assessed using paired t-tests.

(F) Quantitative analysis of Cleaved Caspase-3 protein levels normalized to the Control group, with significance evaluated using paired t-tests (***$p < 0.001$).

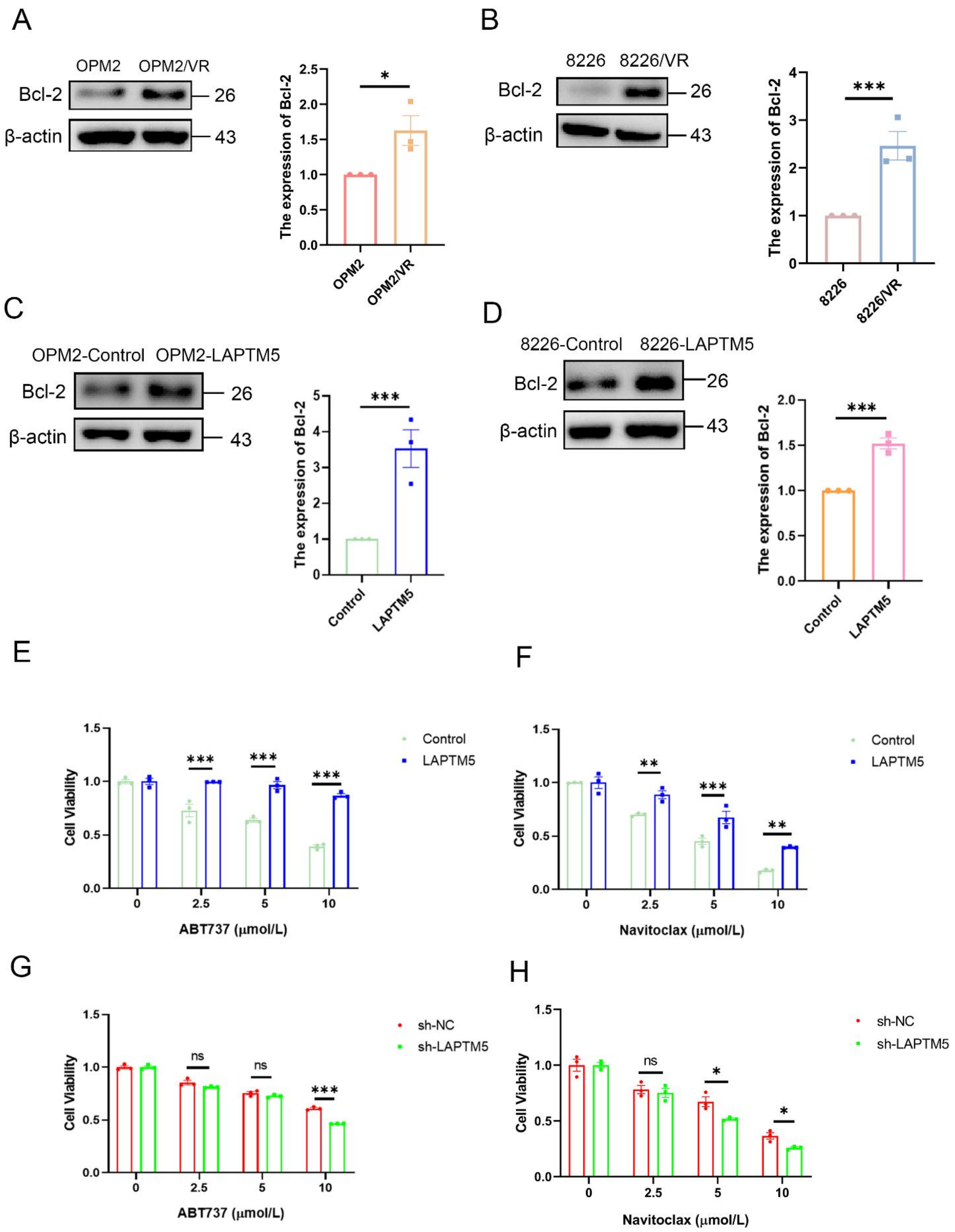
A
OPM2
OPM2/VR
Bcl-2
β-actin
26
43
The expression of Bcl-2
*
B
8226
8226/VR
Bcl-2
β-actin
26
43
The expression of Bcl-2
***
C
OPM2-Control
OPM2-LAPTM5
Bcl-2
β-actin
26
43
The expression of Bcl-2
***
Control
LAPTM5
D
8226-Control
8226-LAPTM5
Bcl-2
β-actin
26
43
The expression of Bcl-2
***
Control
LAPTM5
E
Cell Viability
Control
LAPTM5
ABT737 (μmol/L)
F
Cell Viability
Control
LAPTM5
Navitoclax (μmol/L)
G
Cell Viability
sh-NC
sh-LAPTM5
ns
***
ABT737 (μmol/L)
H
Cell Viability
sh-NC
sh-LAPTM5
ns
*
Navitoclax (μmol/L)

Supplementary Figure S5. LAPTM5 mediated Venetoclax Resistant Is Dependent on Bcl-2

(A) Western blot analysis of Bcl-2 protein expression in OPM2 and OPM2/VR cell lines, with quantitative results normalized to the OPM2 group. Statistical significance was assessed using paired t-tests (*$p < 0.05$).

(B) Western blot analysis of Bcl-2 protein expression in 8226 and 8226/VR cell lines, with quantitative results normalized to the 8226 group. Statistical significance was assessed using paired t-tests (***$p < 0.001$).

(C) Western blot analysis of Bcl-2 protein expression in OPM2-Control and OPM2-LAPTM5 cell lines, with quantitative results normalized to the Control group (***$p < 0.001$).

(D) Western blot analysis of Bcl-2 protein expression in 8226-Control and 8226-LAPTM5 cell lines, with quantitative results normalized to the Control group (***$p < 0.001$).

(E) Cell viability of OPM2-Control and OPM2-LAPTM5 cells treated with ABT-737 (0 μM, 2.5 μM, 5 μM, 10 μM) for 48 hours was measured using the CCK-8 assay (***$p < 0.001$).

(F) Cell viability of OPM2-Control and OPM2-LAPTM5 cells treated with Navitoclax (0 μM, 2.5 μM, 5 μM, 10 μM) for 48 hours, assessed via CCK-8 assay (**$p < 0.01$).

(G) Cell viability of OPM2/VR-shNC and OPM2/VR-sh-LAPTM5 cells treated with ABT-737 (0 μM, 2.5 μM, 5 μM, 10 μM) for 48 hours, determined using the CCK-8 assay (***$p < 0.001$).

(H) Cell viability of OPM2/VR-shNC and OPM2/VR-sh-LAPTM5 cells treated with Navitoclax (0 μM, 2.5 μM, 5 μM, 10 μM) for 48 hours, measured via CCK-8 assay (*$p < 0.05$).

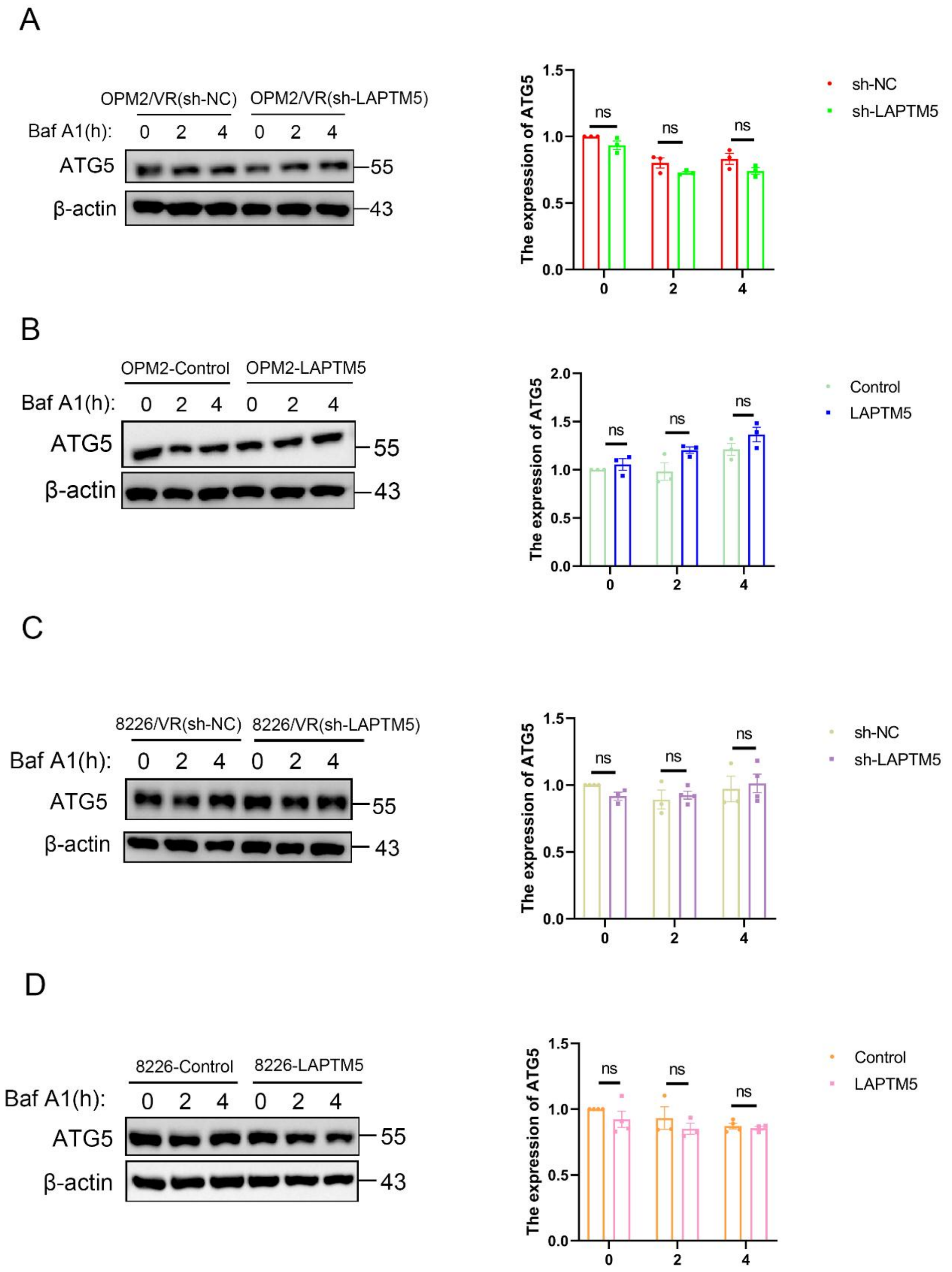

Supplementary Figure S6. LAPTM5 Does Not Regulate ATG5 expression

(A) Western blot analysis of ATG5 protein levels in OPM2/VR-shNC and OPM2/VR-sh-LAPTM5 cells treated with 1 μM BafA1 for 0, 2, and 4 hours. Data are presented as mean ± SEM from three independent experiments, normalized to the sh-NC

group.

(B) Western blot analysis of ATG5 protein expression in OPM2-Control and OPM2-LAPTM5 cells treated with 1 μM BafA1 for 0, 2, and 4 hours, with quantitative data normalized to the Control group.

(C) Western blot analysis of ATG5 protein levels in 8226/VR-sh-NC and 8226/VR-sh-LAPTM5 cells treated with 1 μM BafA1 for 0, 2, and 4 hours. Data are presented as mean ± SEM, normalized to the sh-NC group.

(D) Western blot analysis of ATG5 protein expression in 8226-Control and 8226-LAPTM5 cells treated with 1 μM BafA1 for 0, 2, and 4 hours, with data normalized to the Control group.

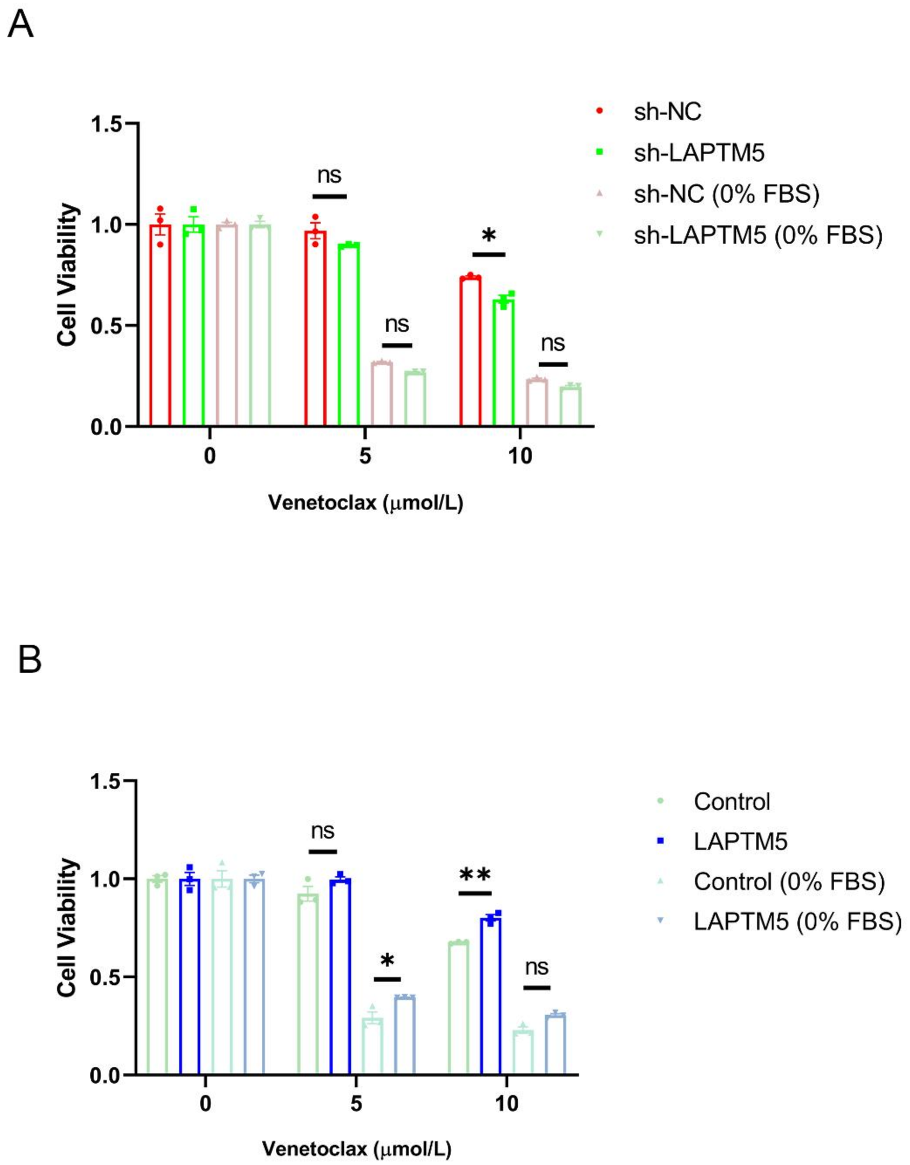

Supplementary Figure S7. LAPTM5-mediated Venetoclax Resistance is Regulated by Nutrient Availability

(A) Cell viability of OPM2/VR-shNC and OPM2/VR-shLAPTM5 cells cultured in normal or serum-free medium for 24 hours and subsequently treated with venetoclax (0 μM, 5 μM, 10 μM) for 48 hours, assessed via the CCK-8 assay (* $p < 0.05$).

(B) Cell viability of OPM2-Control and OPM2-LAPTM5 cells cultured in normal or serum-free medium for 24 hours and treated with venetoclax (0 μM, 5 μM, 10 μM) for 48 hours, determined using the CCK-8 assay (** $p < 0.01$).
